# Can dorzolamide/timolol-fixed combination effectively treat primary open-angle glaucoma? A protocol for systematic review and meta-analysis

**DOI:** 10.1097/MD.0000000000023245

**Published:** 2020-11-20

**Authors:** Yan-Xiu Qi, Hong-wei Liu, Quan Sun, Xing-jie Su, Lin Han

**Affiliations:** a Department of Ophthalmology, First Affiliated Hospital of Jiamusi University; b Department of Biology Laboratory, Jiamusi University School of Basic Medicine; c Department of Otorhinolaryngology, The 163th Hospital of the People's Liberation Army Joint Service Support Force, Jiamusi, China.

**Keywords:** primary open-angle glaucoma, dorzolamide, timolol, efficacy, safety

## Abstract

**Background:**

Primary open-angle glaucoma (POAG) is a very common disorder, and it is the second leading cause that results in blindness worldwide after cataracts. Previous studies have reported that dorzolamide/timolol-fixed combination (DTFC) can be used in treating POAG. However, there are still inconsistent results. Thus, this study will systematically investigate the efficacy and safety of DTFC on POAG.

**Methods:**

A comprehensive search will be carried out in Cochrane Library, MEDLINE, EMBASE, CINAHI, ACMD, China National Knowledge Infrastructure, and WANGFANG database from origin to the present. There are no limitations related to the language and publication status. Only randomized controlled trials that assessed the efficacy and safety of DTFC for the treatment of POAG will be included. Two researchers will independently undertake record selection, data extraction, and study quality assessment. Any divisions will be solved by discussion with a third researcher. We will perform statistical analysis using RevMan 5.3 software

**Results:**

This study will summarize the present evidence to identify the efficacy and safety of DTFC in treating POAG through mean intraocular pressure, best corrected visual acuity, contrast sensitivity, bioelectric activity of the retina, rate of progression of glaucoma, quality of life, and adverse events.

**Conclusions:**

The results of this study will provide evidence of DTFC for the treatment of POAG.

**Systematic review registration:**

INPLASY202040120.

## 1 Introduction

Primary open-angle glaucoma (POAG) is a chronic, progressive, and anterior optic neuropathy that is characterized by increased intraocular pressure (IOP), cupping and atrophy of the optic nerve head, and typical visual field defects.^[1–3]^ It is the leading cause of irreversible visual impairment worldwide,^[4,5]^ and if left untreated, it can ultimately result in severe or complete vision loss.^[6]^ The prevalence of POAG is estimated between 1.5% and 2% in the USA, with most cases detected over 40 years old.^[7,8]^ It has been estimated that the global number of POAG is about 44 million cases in 2013, and will reach to 53 million by 2020.^[9]^ There are several known risk factors that result in POAG, such as increased IOP, advanced age, race, decreased corneal thickness, family history, diabetes, and myopia.^[10–12]^

POAG is associated with high IOP,^[13]^ which leads to degeneration of the optic nerve.^[13,14]^ Interventional treatments, including medical interventions, laser trabeculoplasty and surgery aim at lowering IOP with the target of delaying or halting the progression of POAG.^[15–17]^ Of those, topical medical therapy is the main therapy, and a single topical hypotensive drug is the first line approach. However, more than 40% patients require more than one medication to reach IOP reduction.^[18]^ Fortunately, the fixed combination of single medication is reported to resolve this problem. The dorzolamide/timolol-fixed combination (DTFC) is commonly-prescribed fixed combinations for POAG that has been approved in several countries.^[19,20]^ Dorzolamide is a non-bacteriostatic sulfonamide derivative and topical carbonic anhydrase inhibitor that manages evaluated IOP and relevant ocular hypertension.^[21]^ Timolol is a beta-blocker, which decreases IOP by reducing the production of fluid.^[22]^ DTFC exerts better efficacy than any single medication. Studies suggested that DTFC could help decrease IOP significantly in patients with POAG.^[23–31]^ However, no systematic review has investigated the efficacy and safety of DTFC in treating POAG. Therefore, this study will systematically and comprehensively assess the efficacy and safety of DTFC for the management of POAG.

### 1.1 Aim

This systematic review aims to appraise the efficacy and safety of DTFC for POAG.

### 1.2 Objective

The objective of this systematic review is to comprehensively and systematically search eligible studies and to summarize all available evidence on investigating the efficacy and safety of DTFC compared to other interventions for POAG.

## 2 Methods/design

### 2.1 Study design

This systematic review was registered on INPLASY202040120. It has been reported according to the guidelines of Preferred Reporting Items for Systematic Reviews and Meta-analysis (PRISMA) Protocol and checklist (additional file 1).^[32]^ In brief, this study will be performed in 4 steps:

1. multiple literature sources will be searched to examine relevant records;
2. titles, abstracts, and full-text identifying will be carried out in accordance with predefined eligibility criteria;
3. all essential data will be extracted; and
4. a recommended study quality assessment tool will be utilized to appraise study quality before a meta-analysis will be pursued.

### 2.2 Eligibility criteria

This study consists of following inclusion criteria:

1. only randomized controlled trials (RCTs) will be eligible if they assess the efficacy and safety of DTFC alone in patients with POAG which meet the criteria;
2. we will include all RCTs involving participants with a confirmed diagnosis of POAG in spite of country, race, gender, age, and severity of POAG;
3. RCTs will be included if they randomize participants to utilize DTFC or to other comparators, such as placebo;
4. studies published up to the present in any language and publication status will be included.

The exclusion criteria are presented as follows:

1. all animal study, review, case report, uncontrolled trial, non-RCTs and quasi-RCTs will be excluded;
2. other types of glaucoma will be excluded if they are elaborated clearly, however, any undefined type of glaucoma will be considered for further identifying;
3. glaucoma secondary to other diseases will be excluded;
4. any other treatment combined with DTFC in the experimental group, and any forms of DTFC as comparator in the control group will be excluded in this study; and
5. trials report one of the outcomes of interest.

### 2.3 Outcome measurements

The outcomes of interest for analysis will include primary outcomes of mean intraocular pressure and best corrected visual acuity; and secondary outcomes of contrast sensitivity, bioelectric activity of the retina, rate of progression of glaucoma, quality of life (as assessed by 36-Item Short Form Survey), and adverse events.

### 2.4 Information sources and search method

A comprehensive search will be performed to identify relevant records from origin to the present in the electronic databases of Cochrane Library, MEDLINE, EMBASE, CINAHI, ACMD, China National Knowledge Infrastructure, and WANFANG databases. We will not apply limitations to the language and publication status. We will only consider RCTs that appraised the efficacy and safety of DTFC for POAG. We have summarized search strategy sample for Cochrane Library (Table 1), and will create similar search strategies for other electronic databases. The search terms include “glaucoma”, “intraocular pressure”, “ocular hypertension”, “intraocular hypertension”, “open-angle”, “primary”, “optic neuropathy”, “timolol”, “Timoptic”, “Istalol”, “Timoptic-xe”, “dorzolamide”, “Trusopt”, “fixed”, “combination”, “randomized controlled trials”, “random”, “randomised”, “randomly”, “allocation”, “placebo”, “blind”, “clinical trials”, and “controlled trials”. In addition, we will scrutinize other sources, such as Google Scholar, conference proceedings, and reference lists of included trials.

**Table 1:** Search strategy used in Cochrane Library database.

Number	Search terms
1	MeSH descriptor: (glaucoma, open-angle) explode all trees
2	((glaucoma^∗^) or (intraocular pressure^∗^) or (ocular hypertension^∗^) or (intraocular hypertension^∗^) or (open-angle^∗^) or (primary^∗^) or (optic neuropathy^∗^)):ti, ab, kw
3	Or 1–2
4	MeSH descriptor: (timolol) explode all trees
5	(dorzolamide) explode all trees
6	((timolol^∗^) or (Timoptic^∗^) or (Istalol^∗^) or (Timoptic-xe^∗^) or (dorzolamide^∗^) or (Trusopt^∗^) or (fixed^∗^) or (combination^∗^)):ti, ab, kw
7	Or 4–6
8	MeSH descriptor: (randomized controlled trials) explode all trees
9	((random^∗^) or (randomised^∗^) or (randomly^∗^) or (allocation^∗^) or (placebo^∗^) or (blind^∗^) or (clinical trials^∗^) or (controlled trials^∗^)):ti, ab, kw
10	Or 8–9
11	3 and 7 and 10

### 2.5 Study selection

All citations will be imported into Endnote X9 and duplicates will be removed. Two researchers (YXQ and HWL) will independently identify titles/abstracts of sought records to eliminate unrelated studies. Then, full papers of potential trials will be obtained and further inspected against all eligibility criteria. If any differences are identified, we will invite a third researcher (QS) to solve them by discussion and a final decision will be reached. Any reasons for excluded studies will be listed. The process of study selection will be reported following PRISMA statement, and will be exerted in a PRISMA flowchart.

### 2.6 Data extraction and management

Two researchers (XJS and LH) will independently extract data from all included RCTs using a predefined, structured and standard data-extraction form. Any divergences will be resolved through discussion with a third researcher (QS). The extracted information consists of study information (e.g., title, first author, year of publication, and country), patient characteristics (e.g., diagnosis criteria, and eligibility criteria), study methods (e.g., sample size, randomization, blind, and concealment); details of intervention and controls (e.g., treatment types, dosage, and duration), outcome measurements, results, findings, follow-up information, adverse events, and conflict of interest. We will express continuous data using means, standard deviations, standard errors with 95% confidence intervals (CIs), and dichotomous data using frequencies or percentages (%) with 95% CIs.

### 2.7 Dealing with missing data

We will contact primary authors to request any unclear or missing data. If it can not be obtained, we will analyze available data using intention-to-treat analysis, and will discuss its possible affects to the study findings.

### 2.8 Risk of bias assessment

Two researchers (YXQ and HWL) will independently appraise methodological quality for all eligible RCTs using Cochrane Handbook for Systematic Reviews of Interventions Tool.^[33]^ It covers 7 aspects, and each item is further rated as “high risk of bias”, “unclear risk of bias” or “low risk of bias”. Any disagreements will be resolved by a third researcher (QS) through consultation and a consensus will be reached.

### 2.9 Quality of evidence rating

Two researchers (XJS and LH) will independently assess the overall strength of the evidence using Grading of Recommendations Assessment, Development and Evaluation tool.^[34]^ It includes study limitations, inconsistency of results, indirectness of evidence, imprecision, and reporting bias.^[34]^ It categorizes the quality of evidence in 1 of 4 levels: high quality, moderate quality, low quality, and very low quality.^[34]^ Its results will be demonstrated in the table of Summary of Findings. A third researcher (QS) will help to solve any disagreements.

### 2.10 Statistical analysis

RevMan 5.3 software (Cochrane, London, UK) will be utilized to perform statistical analysis. In terms of treatment effect measures, mean difference (MD) or standardized MD and 95% CIs will be used for continuous outcomes, and risk ratio and 95% CIs will be utilized for dichotomous outcomes. We will appraise the inconsistencies cross studies using *I*^2^ test.^[35]^ We define *I*^2^ ≤ 50% as having minor heterogeneity, and will use a fixed-effects model to pool the data^[36]^; while *I*^2^ > 50% as having remarkable heterogeneity, and will apply a random-effects model to synthesize the data.^[37]^ If it is possible, we will conduct meta-analysis when minor heterogeneity across sufficient data on outcomes is extracted. If the outcome data can not be pooled, we will present a narrative analysis of individual trials. If sufficient data is available, subgroup analyses will be performed to identify the potential sources of heterogeneity according to the variations in study and patient characteristics, different types of interventions and controls, and different study quality. A sensitivity analysis will be conducted to test the stability of conclusions by eliminating low quality trials. If necessary, we will perform a funnel plot and Egger regression test to check reporting bias when over 10 RCTs are included.

### 2.11 Dissemination and ethics

This study will be published in print, conferences or by peer-reviewed journals. No ethic approval is needed in this study, because it will not use individual data.

### 2.12 Amendments

Any changes to this protocol will be noted with reference to saved searches and analysis.

## 3 Discussion

Fixed combination therapy in treating POAG has gained popularity in recent years. At present, DTFC is the first fixed combination that comprises of dorzolamide and timolol, and is currently routinely utilized in clinical practice in treating POAG. To date, several clinical trials have investigated the efficacy and safety of DTFC for the treatment of POAG, and their findings indicate that DTFC can significantly reduce IOP in patients with POAG.^[23–31]^ To the best of our knowledge, rarely data at systematic level is available on assessing efficacy and safety of DTFC for the treatment of POAG. Thus, this systematic review protocol is the first one to specifically explore the efficacy and safety of DTFC alone in treating POAG. We believe that the findings of this study may provide evidence at evidence-based medicine level to determine whether DTFC is effective and safe for the treatment of POAG. Its results may benefit patients, clinicians, and health-related decision-makers.

On the other hand, this proposed study may still have several limitations. First, the study quality of included trials may be poor. Second, there is insufficient number of included studies and their sample size may be low. Third, remarkable heterogeneity across included trials may exert. All those limitations may affect stability and robustness of the study results, and may result in misleading interpretation of study findings.

## Author contributions

**Conceptualization:** Yan-Xiu Qi, Hong-wei Liu, Quan Sun, Xing-jie Su.

**Data curation:** Yan-Xiu Qi, Hong-wei Liu, Quan Sun, Xing-jie Su, Lin Han.

**Formal analysis:** Hong-wei Liu, Lin Han.

**Funding acquisition:** Hong-wei Liu.

**Investigation:** Hong-wei Liu.

**Methodology:** Yan-Xiu Qi, Quan Sun, Xing-jie Su.

**Project administration:** Hong-wei Liu.

**Resources:** Yan-Xiu Qi, Quan Sun, Xing-jie Su, Lin Han.

**Software:** Yan-Xiu Qi, Quan Sun, Xing-jie Su, Lin Han.

**Supervision:** Hong-wei Liu.

**Validation:** Yan-Xiu Qi, Hong-wei Liu, Lin Han.

**Visualization:** Yan-Xiu Qi, Hong-wei Liu, Quan Sun, Xing-jie Su, Lin Han.

**Writing – original draft:** Yan-Xiu Qi, Hong-wei Liu, Quan Sun, Xing-jie Su, Lin Han.

**Writing – review & editing:** Yan-Xiu Qi, Hong-wei Liu, Quan Sun, Xing-jie Su.
